# Supplementary material for: Heat-assisted hot-hole transfer increases the surface-enhanced Raman activity of Au-TiO2 nanoarrays
Source: Nat Commun. 2026 Mar 17;17:4047. doi: 10.1038/s41467-026-70822-4 (PMC13139474; doi:10.1038/s41467-026-70822-4)
Supplement: Supplementary file 1 — Supplementary Information [file 41467_2026_70822_MOESM1_ESM.pdf]

Supplementary Information for

Heat-assisted hot-hole transfer increases the surface-enhanced Raman activity of Au-TiO_2 nanoarrays

Mengya Zhang[1†], Tongcheng Yu[1,2†], Hao Liu[1,2], Chao Lin[1], Yaping Yang[3], Bowen Lv[1], Qi Zhang[1,4*], Ming Chen[5*], Tianshuai Wang[6], Weihong Hua[1,2], and Kai Han[1,2*]

[1]*College of Advanced Interdisciplinary Studies, National University of Defense Technology, Changsha, 410073, China.*

[2]*Nanhu Laser Laboratory, National University of Defense Technology, Changsha, 410073, China.*

[3]*College of Aerospace Science and Engineering, National University of Defense Technology, Changsha, 410073, China.*

[4]*Department of Applied Physics, Nanjing University of Science and Technology, Nanjing, Jiangsu 210094, China.*

[5]*School of Physics, Shandong University, Jinan 250100, Shandong, China.*

[6]*School of Chemistry and Chemical Engineering, Northwestern Polytechnical University, Xi'an, 710129, China.*

[†]These authors contributed equally to this work.

*To whom correspondence should be addressed: *zhangqi24@njust.edu.cn*; *chenming@sdu.edu.cn*; *hankai0071@nudt.edu.cn*

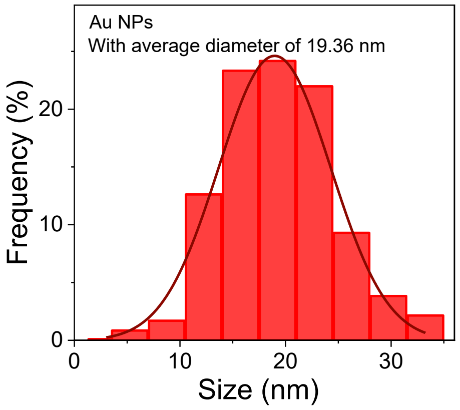

Supplementary Figure 1. The size distribution of Au NPs in Au-TiO_2 NAs, which was obtained by measuring the diameter of more than 300 Au NPs on a SEM image. Au NPs have a size distribution at around 19 nm.

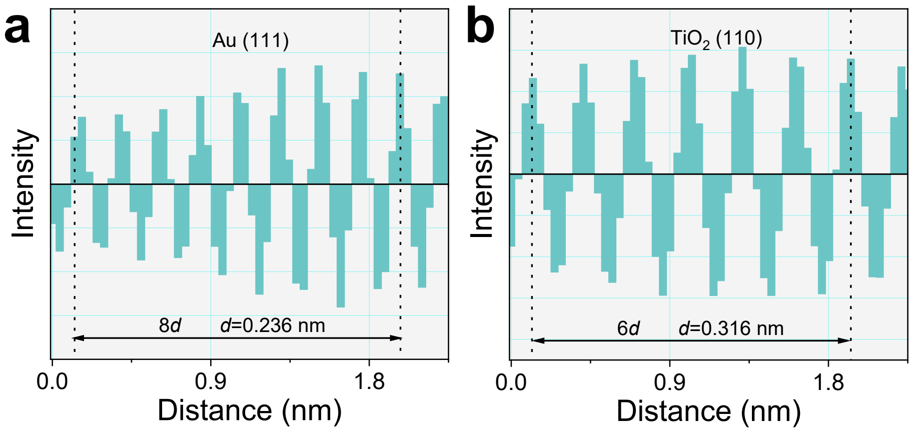

Supplementary Figure 2. The corresponding lattice fringes characterized in the HRTEM image of Au in **a**, and TiO_2 in **b**. Source data are provided as a Source Data file.

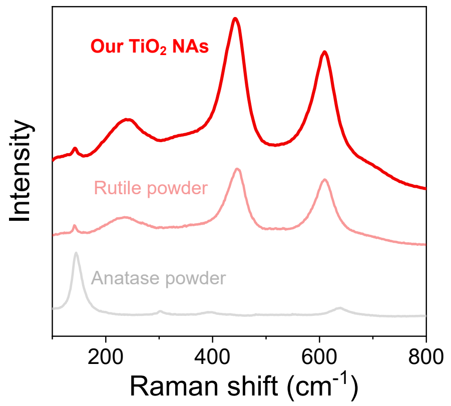

Supplementary Figure 3. Raman spectrum of our TiO_2 NA substrates as well as anatase TiO_2 powder (aladdin 99.9%) and rutile TiO_2 powder (aladdin 99.9%). Four major peaks that represent A_{1g}, E_g, the second-order effect (SOE), and B_{1g} Raman-active vibrational modes are located at 609, 446, 235, and 141 cm^{-1} respectively, indicating that the predominant phase of the TiO_2 NAs is rutile. Source data are provided as a Source Data file.

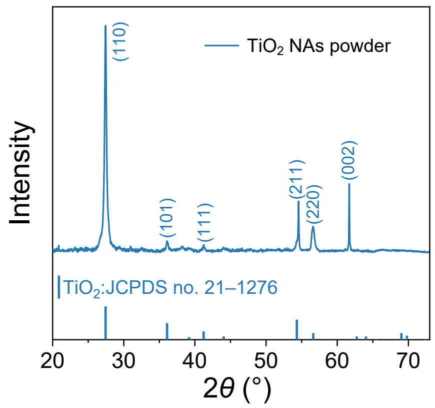

Supplementary Figure 4. XRD patterns of TiO_2 NAs powder. To confirm that the absence of the (110) peak was due to the vertical alignment, we removed TiO_2 NAs powder from the FTO substrate via intensive ultrasound for XRD testing, which showed the intense (110) peak of the reference pattern of rutile at ≈27°. Source data are provided as a Source Data file.

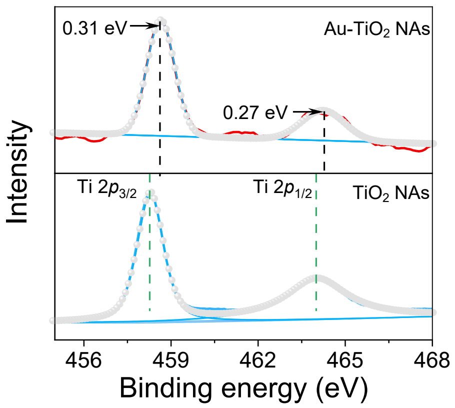

Supplementary Figure 5. Analysis of chemical state and synergistic coupling effect of Au-TiO_2 NAs. XPS fine spectra of Ti 2*p* originated from original TiO_2 NAs and as-prepared Au-TiO_2 NAs. The XPS peaks of Ti 2*p* at 458.3 eV and 464.0 eV are assigned to Ti $2p_{3/2}$ and Ti $2p_{1/2}$[1,2]. Compared with original TiO_2 NAs, the XPS spectroscopy indicates that Ti 2*p* of Au-TiO_2 NAs showed a shift in binding energy. Source data are provided as a Source Data file.

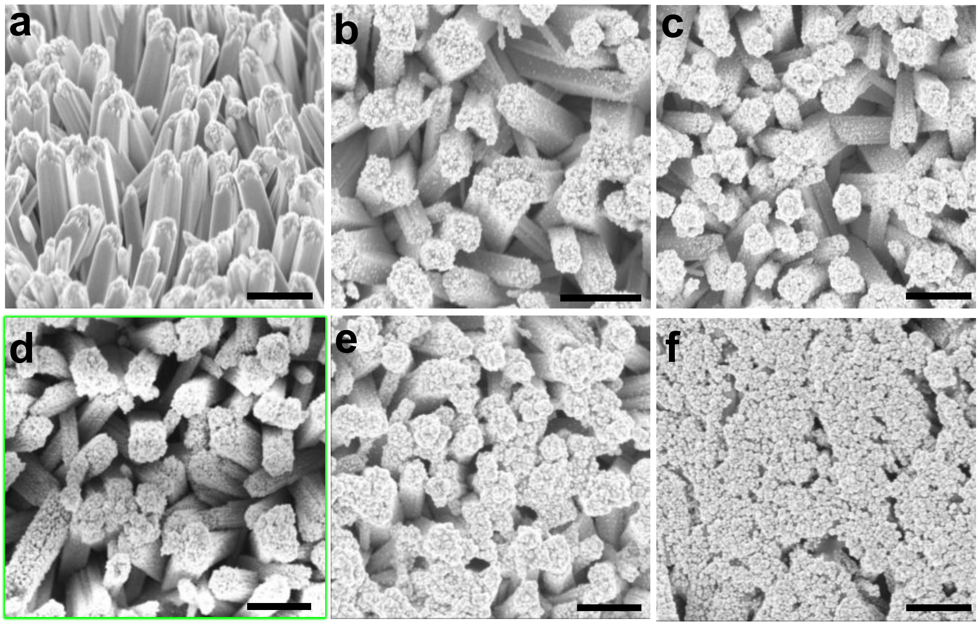

Supplementary Figure 6. SEM images of **a** original TiO_2 NAs and a series of Au-TiO_2 products with different irradiation time of 375 nm laser: **b**: 5 min; **c**:15 min; **d**: 25 min; **e**: 35 min; **f**: 45 min, and the scale bar in both figures is 500 nm.

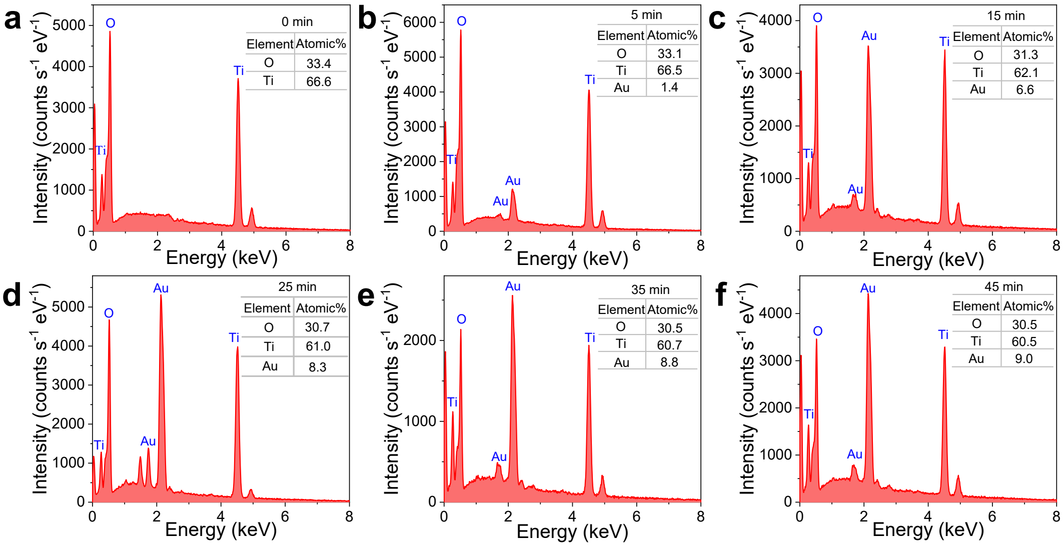

Supplementary Figure 7. Composition analysis. **a-f** The corresponding EDS patterns of original TiO_2 NAs and a series of Au-TiO_2 products with different irradiation time. Source data are provided as a Source Data file.

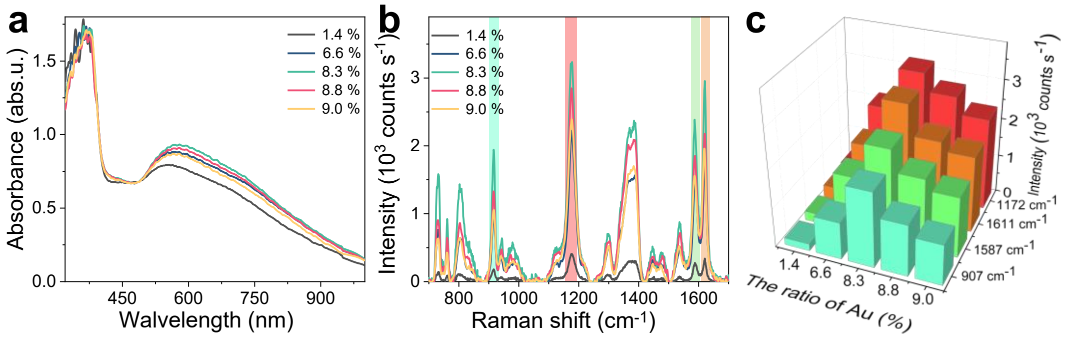

Supplementary Figure 8. Optical properties and SERS activities of Au-TiO_2 products. **a** The absorption

spectrums of a series of Au-TiO_2 products with different Au content, and the unit (abs.u.) on the y-axis is equivalent to optical density (OD); **b** The SERS activities of 10^{-7} M CV on a series of Au-TiO_2 substrates with different Au content (tested at room temperature); **c** The corresponding variation of the four main Raman peak intensities of CV molecules at Au-TiO_2 substrates with different Au content. Source data are provided as a Source Data file.

Supplementary Note 1. Selection of the optimal substrates.

The typical microstructures of as-prepared original TiO_2 NAs and a series of Au-TiO_2 products were characterized by SEM. As displayed in Supplementary Fig. 6, the original TiO_2 NAs were uniformly and orderly oriented coated on the surface of FTO with an average diameter of 100–200 nm and height of ≈1.2 μm. The SEM image reveals that there are gaps between the original TiO_2 NAs, possessing a comparatively huge specific surface area to support enough space for the growth of Au NPs. Meanwhile, the LSPR effect is strongly dependent on the morphology and density of the Au NPs[2,3], so we optimized different metal components of Au by simply adjusting the irradiation time. After UV laser irradiation for 5–15 min, a small number of Au NPs gradually grew on the TiO_2 NAs, and after 25 min, a large number of interconnected Au NPs were uniformly distributed on the top and sides of the TiO_2 NAs. However, if the irradiation time was extended to 35–45min, excessive Au NPs gradually covered the surface of the array. The EDS results also demonstrated the above process, as the portion of Au increased in Supplementary Fig. 7.

The tunable optical and LSPR characteristics of Au-TiO_2 NAs were confirmed by UV–VIS absorption spectra, and SERS spectra. The absorption at ≈580 nm was drastically enhanced with an increase of irradiation time (5–25 min), which is owing to the increased Au NPs loading amount (1.4%–8.3%) on the TiO_2 surfaces (Supplementary Fig. 8a). However, the excessive irradiation time (35–45 minutes) caused a large amount of Au NPs (8.8%–9.0%) to accumulate on top of the TiO_2 nanoarray, which strongly prevented light from reaching the interior of the nanoarray, resulting in a reduction in the light absorption capacity of Au-TiO_2 NAs. Thus, the Au-TiO_2 NAs with 8.3% Au (25 min) have the highest light-harvesting efficiency.

On the other hand, we further characterized the LSPR properties of a series of Au-TiO_2 products through their different SERS performance. The SERS activities of these corresponding structures were assessed by using CV (10^{-7} M) as probe molecule. As shown in Supplementary Fig. 8b and c, Au-TiO_2 heterostructures with 8.3% Au composition provide maximally enhanced SERS activity. However, the SERS signal decreases with further prolonging irradiation time to 45 min, which is related to the excessive aggregations of Au NPs reduced the surface area of Au-TiO_2 contacting with the photo flux and the probe molecule. These results also imply that the highly dense and uniform Au NPs (8.3% Au) loaded on TiO_2 NR supports can provide excellent LSPR properties, which contributes to improve SERS activity.

We report an attractive Au-TiO_2 NAs substrates with efficient hot carriers transport ability and excellent chemical, mechanical stability[1,4,5]. Our Au-TiO_2 NAs possess several inherent advantages: (1) The vertically arranged TiO_2 NAs have a high specific surface area[4,6], which provides sufficient active sites for the loading of Au NPs to realize the controllable growth of dense and evenly distributed Au NPs on TiO_2 NAs (Fig. 1b). (2) Additionally, owing to multiple light-scattering effects of the TiO_2 NAs and the strong LSPR effect of the large quantity of Au NPs[6], the Au-TiO_2 NAs exhibit an excellent light harvesting ability. (3) Moreover, the vertically aligned Au-TiO_2 NAs allow the directional electron transport, and the formation of Schottky barrier at the interface can effectively promote charge separation[4], both of which facilitate the efficient transfer of plasmon-

induced hot carriers from Au NPs to neighboring TiO_2 NAs. This makes the Au-TiO_2 NAs substrate grasp great potential in providing efficient SERS chemical enhancement.

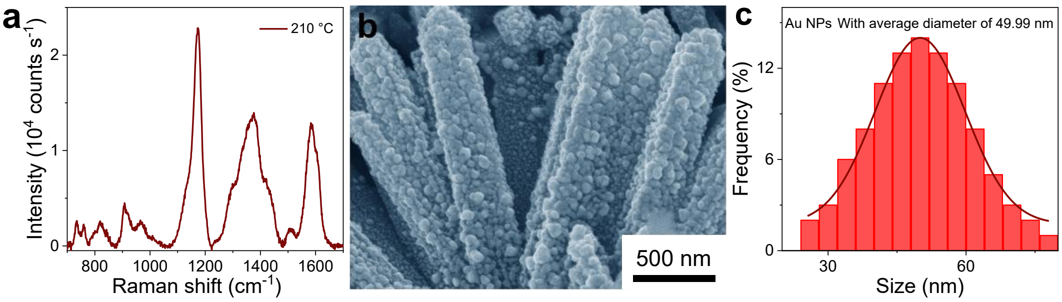

Supplementary Figure 9. SERS and structure changes caused by excessive temperature. **a** The SERS spectra recorded for CV (10^{-7} M) on Au-TiO_2 NAs with temperature of 210 °C, showing only a 7.06-fold intensity enhancement (at 1172 cm^{-1}) compared to that at 22 °C, which is lower than the 11.41-fold enhancement observed at 180 °C; **b** The SEM images of the as-fabricated Au-TiO_2 NAs after heating at 210 °C for 10 min; **c** The corresponding size distribution of Au NPs. The microstructure of Au-TiO_2 NAs was changed under excessive high temperature (210 °C), resulting in a decrease in SERS activity compared to 180 °C. Source data are provided as a Source Data file.

Supplementary Table 1. Key Raman resonances and their enhancement.

	v_i Raman (cm^{-1})	Intensity (counts s^{-1})	v_i 22 °C-SERS (cm^{-1})	Intensity (counts s^{-1})	AEF	v_i 180 °C-TI-SERS (cm^{-1})	Intensity (counts s^{-1})	AEF	TI-SERS /SERS
CV	731	48	731	1583	6.60×10^7	735	5681	2.37×10^8	3.59
	908	75	916	1945	5.19×10^7	907	5533	1.48×10^7	2.84
	1175	78	1178	3236	8.30×10^7	1172	36916	9.47×10^8	11.41
	1589	74	1587	2386	6.45×10^7	1587	19163	5.18×10^8	8.03
	1622	91	1621	2956	6.57×10^7	1611	14179	3.15×10^8	4.80
MB	766	50	770	1299	5.18×10^7	768	8997	3.59×10^8	6.93
	1056	37	1041	487	2.60×10^7	1039	3964	2.12×10^8	8.15
	1168	51	1182	883	3.50×10^7	1181	4309	1.71×10^8	4.88
	1387	107	1397	2212	4.13×10^7	1395	14265	2.67×10^8	6.45
	1611	153	1624	2646	3.46×10^7	1622	20551	2.69×10^8	7.77
Glibenclamide	800	44	804	287	3.27×10^5	802	1617	1.84×10^6	5.63
	937	159	937	828	2.61×10^5	934	1013	3.19×10^5	1.22
	1153	40	1158	329	4.10×10^5	1161	2386	2.97×10^6	7.25
	1238	24	1245	329	6.94×10^5	1243	1368	2.89×10^6	4.16
	1595	67	1601	393	2.95×10^5	1594	1831	1.37×10^6	4.66
Metformin hydrochloride	733	108	748	1311	2.43×10^6	748	3349	6.20×10^6	2.55
	933	94	934	1058	2.25×10^6	936	1666	3.54×10^6	1.57
	1088	19	1073	626	6.56×10^6	1072	3153	3.30×10^7	5.04
	1285	15	1287	3206	4.35×10^7	1287	13710	1.86×10^8	4.28
Thiamphenicol	679	30	691	603	1.00×10^6	709	3643	6.04×10^6	6.05
	1077	26	1078	1424	2.72×10^6	1074	2948	5.63×10^6	2.07
	1145	36	1145	1609	2.22×10^6	1144	4021	5.54×10^6	2.50
	1590	49	1603	4890	4.99×10^6	1591	16031	1.63×10^7	3.28
Streptomycin	707	64	698	1267	3.97×10^6	685	5179	1.63×10^7	4.09
	855	103	850	2832	5.51×10^6	835	5668	1.10×10^7	2.00
	943	237	937	2261	1.91×10^6	937	5015	4.23×10^6	2.22
	1018	111	1004	1749	3.15×10^6	1013	3559	6.40×10^6	2.04
Folic acid	1353	90	1378	2320	5.14×10^6	1375	4789	1.06×10^7	2.06
	1568	90	1574	918	2.04×10^6	1575	3827	8.51×10^6	4.17
	1604	170	1603	1381	1.63×10^6	1593	5517	6.50×10^6	4.00
Thiram	570	24	571	3353	2.84×10^7	571	10436	8.83×10^7	3.11
	852	22	872	1113	1.03×10^7	873	2337	2.17×10^7	2.10
	1150	8	1147	4145	1.02×10^8	1142	9512	2.34×10^8	2.29
	1377	19	1382	12293	1.30×10^8	1373	28126	2.97×10^8	2.29
	1460	10	1451	2696	5.55×10^7	1443	6481	1.33×10^8	2.40
Penicillin G Sodium	1005	135	1006	3142	4.66×10^5	1006	6720	9.97×10^5	2.14
	1203	14	1219	634	9.31×10^5	1217	2360	3.46×10^6	3.72

	1292	16	1290	972	1.24×10^6	1288	2991	3.80×10^6	3.08
	1586	12	1567	687	1.15×10^6	1562	3751	6.26×10^6	5.46

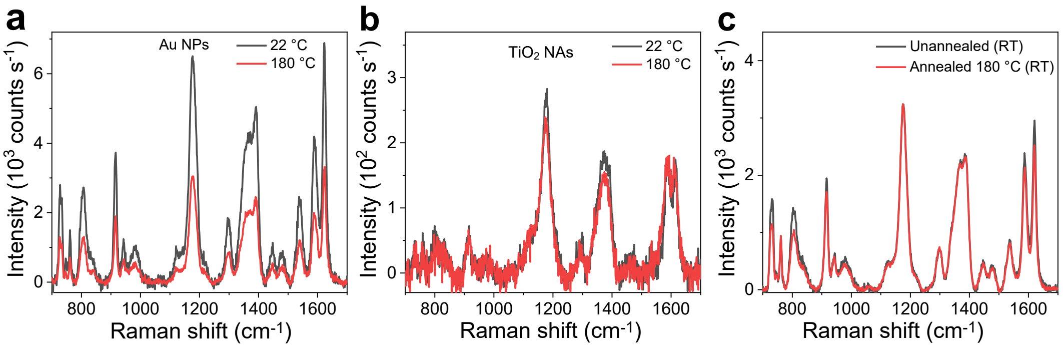

Supplementary Figure 10. Control experiments. **a** Raman spectra of 10^{-5} M CV molecules separately performed at 22 °C and 180 °C based on Au NPs substrate; **b** Raman spectra of 10^{-3} M CV molecules separately performed at 22 °C and 180 °C based on TiO_2 NAs substrate; **c** The Au-TiO_2 NAs were annealed at 180 °C in an oven for 70 min. At room temperature, Raman spectra (10^{-7} M CV) revealed no SERS enhancement was observed for the 180 °C-annealed Au-TiO_2 NAs (annealed 180 °C, RT) compared with the pristine samples (unannealed, RT). Source data are provided as a Source Data file.

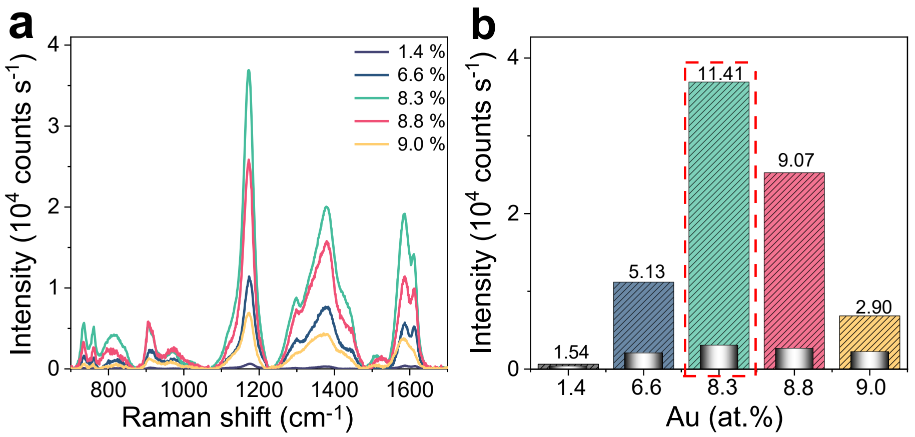

Supplementary Figure 11. a TI-SERS spectra of 10^{-7} M CV molecules adsorbed on a series of Au-TiO_2 products with different Au contents under 180 °C; **b** Comparison of the corresponding TI-SERS (solid bars) and SERS (hatched bars) signal intensities of a series of Au-TiO_2 products at 1172 cm^{-1}, and the numbers above the bars indicate the enhancement factor of TI-SERS relative to conventional SERS. Source data are provided as a Source Data file.

Supplementary Note 2. Comparative study of the high temperature SERS performance of different substrates.

To further demonstrate the TI-SERS effect, the control tests were conducted using Au NPs and original TiO_2 NAs. As plotted in Supplementary Fig. 10, we performed high-temperature SERS tests based on Au NPs, and original TiO_2 NAs substrate using CV molecules, where no significant SERS enhancement was observed under 180 °C heating. Moreover, the Au-TiO_2 NAs are replaced by other Au-TiO_2 complexes with different components to verify the universal adaptation of TI-SERS. As shown in Supplementary Fig. 11, the TI-SERS activity based on other Au-TiO_2 complexes can be also enhanced by the high temperature-excited strategy. This significant comparison confirmed that it is the interaction between Au and TiO_2 of the Au-TiO_2 complexes under high temperature play an important role in the enhancement of TI-SERS.

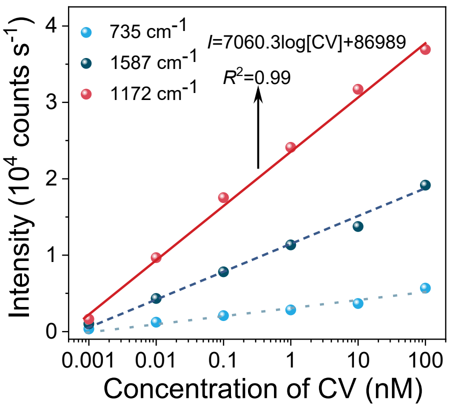

Supplementary Figure 12. Detection of CV molecules. The variations of TI-SERS characteristic peak intensities versus the CV concentrations. The Raman peak intensities display reasonable linear responses to the change of CV concentration on the logarithmic scale: I=7060.3log[CV]+86989 (units for both constants are counts s^{-1}). It is beneficial for quantitative and ultra-trace monitoring of pollutants in practical applications. Source data are provided as a Source Data file.

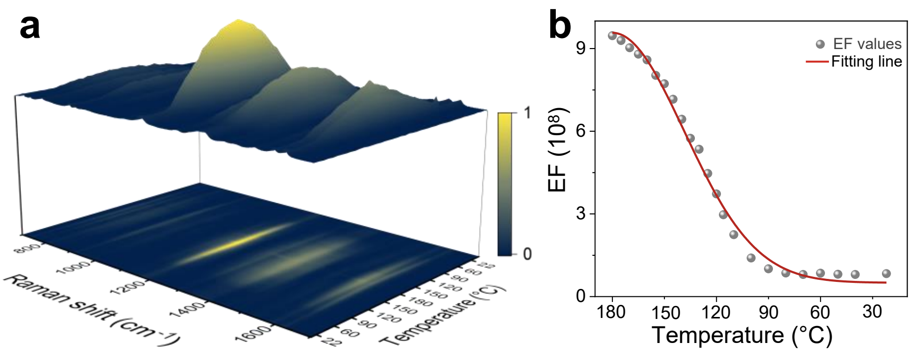

Supplementary Figure 13. a The TI-SERS spectra recorded from CV (10^{-7} M) on Au-TiO_2 NAs with temperature from 22 °C to 180 °C; **b** The EF values of the characteristic peak at 1172 cm^{-1} change with temperature (during colling), red line shows the trend of the signal changes. The decay of EF during temperature decreases still followed the same quantitative relationship as at heating ($EF \propto e^{-(T-180\ °C)^2}$), demonstrating the tunable property of the enhancement factor through temperature regulation. Source data are provided as a Source Data file.

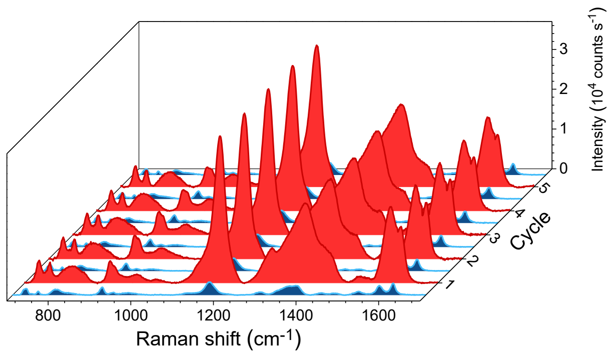

Supplementary Figure 14. The SERS spectra comparison at the thermal field of 180 °C and no extra thermal field of 22 °C at five consecutive cycles. Source data are provided as a Source Data file.

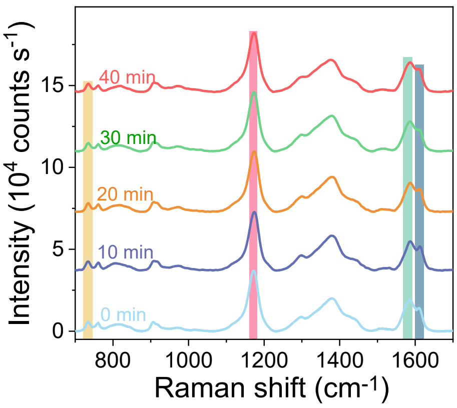

Supplementary Figure 15. TI-SERS spectra of Au-TiO_2 NAs substrate at 180 °C varied with time from 0 to 40 min. Source data are provided as a Source Data file.

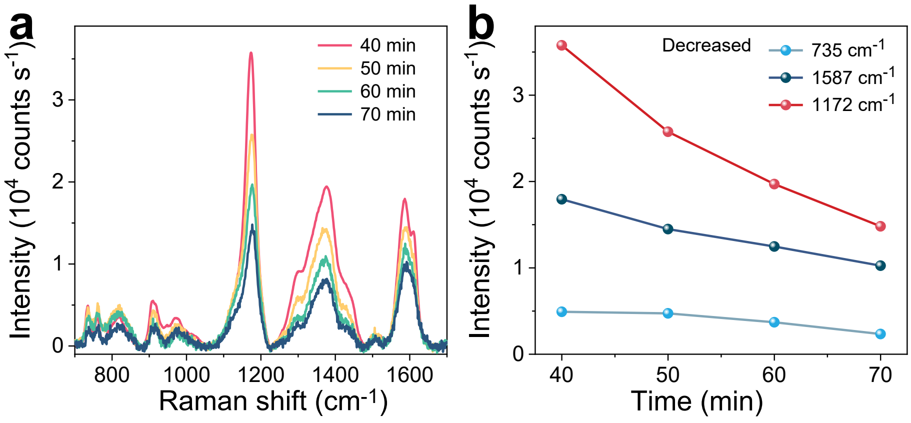

Supplementary Figure 16. Thermal degradation of probe molecules. **a** TI-SERS spectra changed with time (40−70 min) of Au-TiO_2 substrate at 180 °C; **b** Corresponding changes of SERS signal intensities at 735, 1587, 1172 cm^{-1}. The prolonging time (50−70 min) led to a gradual weakening of SERS signals, which is caused by thermal degradation of the probe molecules[7]. Source data are provided as a Source Data file.

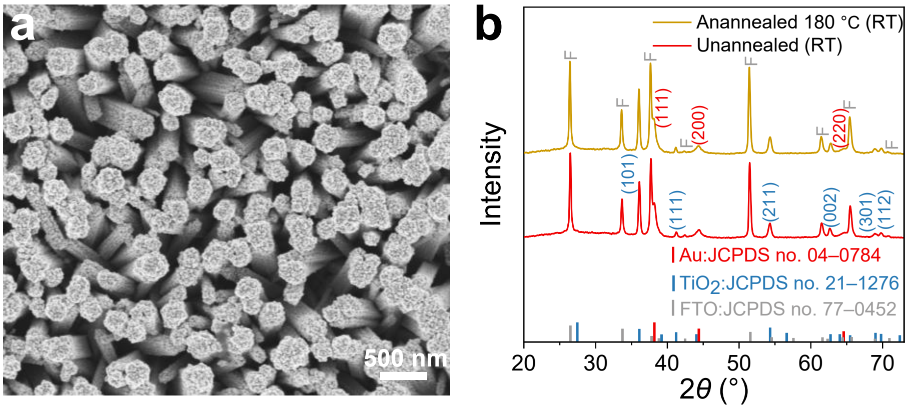

Supplementary Figure 17. Stability of Au-TiO_2 NAs structure at high temperature. **a** The SEM image and **b** XRD patterns of the prepared Au-TiO_2 NAs after heating at 180 °C for 70 min, measured at room temperature. The SEM and XRD results show that the morphology and crystallinity of the annealed Au-TiO_2 NAs have not undergone notable changes compared to the original samples, indicating the structure of the Au-TiO_2 NAs remains stable after heating at 180 °C. Source data are provided as a Source Data file.

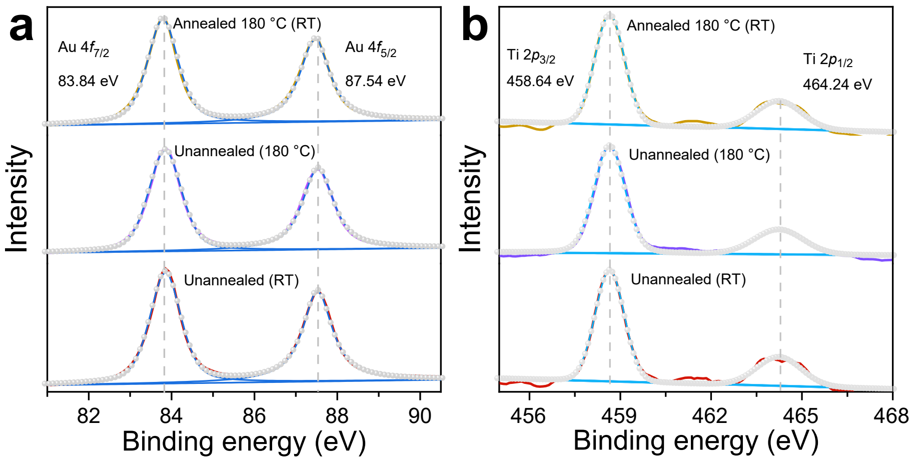

Supplementary Figure 18. a–b XPS spectra of the unannealed Au-TiO_2 NAs tested at room temperature (unannealed, RT) and at 180 °C (unannealed, 180 °C), as well as the 180 °C-annealed Au-TiO_2 NAs tested at room temperature (annealed 180 °C, RT). Source data are provided as a Source Data file.

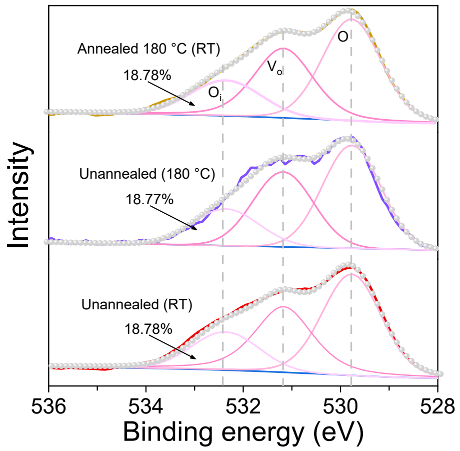

Supplementary Figure 19. O 1*s* XPS spectra of the unannealed Au-TiO_2 NAs tested at room temperature (unannealed, RT) and at 180 °C (unannealed, 180 °C), as well as the 180 °C-annealed Au-TiO_2 NAs tested at room temperature (annealed 180 °C, RT). Source data are provided as a Source Data file.

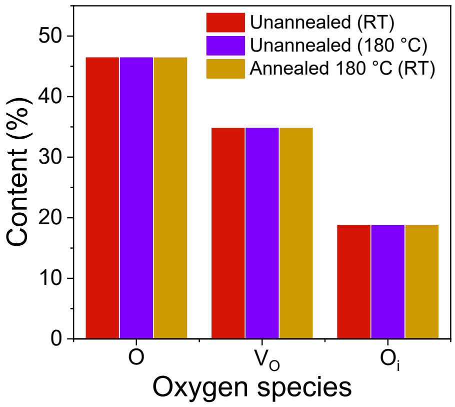

Supplementary Figure 20. Based on Supplementary Fig. 19, we obtained the proportion of O, V_O, and O_i in the unannealed Au-TiO_2 NAs tested at room temperature (unannealed, RT) and at 180 °C (unannealed, 180 °C), as well as the 180 °C-annealed Au-TiO_2 NAs tested at room temperature (annealed 180 °C, RT). Source data are provided as a Source Data file.

Supplementary Note 3. Data analysis of in situ XPS from 22 °C to 180 °C on Au-TiO_2 NAs.

The in situ XPS analysis of Au-TiO_2 NAs at 180 °C (unannealed, 180 °C) was conducted to investigate the changes of the sample during heating, and the XPS test at room temperature of the Au-TiO_2 NAs annealed at 180 °C (annealed 180 °C, RT) was applied to reflect the changes of the samples after heating. As shown in Supplementary Fig. 18, the binding energies of Au 4*f* and Ti 2*p* show no shift during and after heating compared to the pristine Au-TiO_2 NAs (unannealed, 180 °C). What's more, the oxygen defects (oxygen vacancies and incorporation) may influence the charge transfer and band structure of TiO_2 heterojunctions[8-10]. Therefore, the influences of heat treatment on chemical states of O 1*s* were characterized by XPS. As shown in Supplementary Figs. 19 and 20, the XPS pattern of O1*s* can be deconvoluted into three peaks: 529.8 eV assigned to oxygen atoms in TiO_2 (O), 531.2 assigned to the oxygen vacancies (V_O), and 532.4 eV assigned to the additional O species adsorbed on the surface of the sample (O_i). It is obviously that the contents of O, V_O, and O_i remained unchanged during and after heating (Supplementary Figs. 19−20) compared to the pristine Au-TiO_2 NAs, which demonstrates that 180 °C heat treatment cannot induce oxygen-related defects in our substrate.

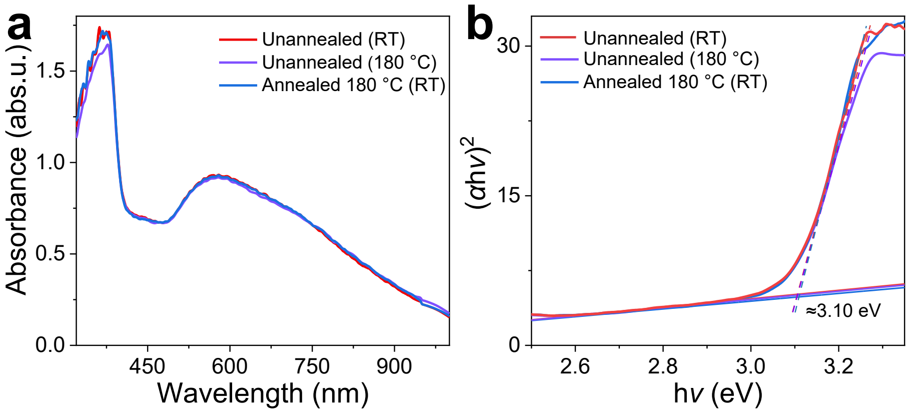

Supplementary Figure 21. a The absorption spectra of the unannealed Au-TiO_2 NAs tested at room temperature (unannealed, RT) and at 180 °C (unannealed, 180 °C), as well as the 180 °C-annealed Au-TiO_2 NAs tested at room temperature (annealed 180 °C, RT); **b** The corresponding Tauc plot analysis for band gap width. Source data are provided as a Source Data file.

Supplementary Note 4. Data analysis of in situ absorption spectra from 22 °C to 180 °C on Au-TiO_2 NAs.

The band gap of Au-TiO_2 NAs was obtained from Tacu plots (Supplementary Fig. 21), which could be determined using the plots of $(\alpha h\nu)^2$ versus photon energy (hν) due to the direct gap of TiO_2[11]. The baseline of the spectra was obtained through linear fitting of the low-energy data points[12,13], and the band gap values are estimated from the intercept of the tangent to the absorption edge with the baseline of the spectra. The absorption spectra and bandgap during and after heating remained consistent with the pristine Au-TiO_2 NAs, indicating that the heat treatment has no influence on the energy band structure of our substrates.

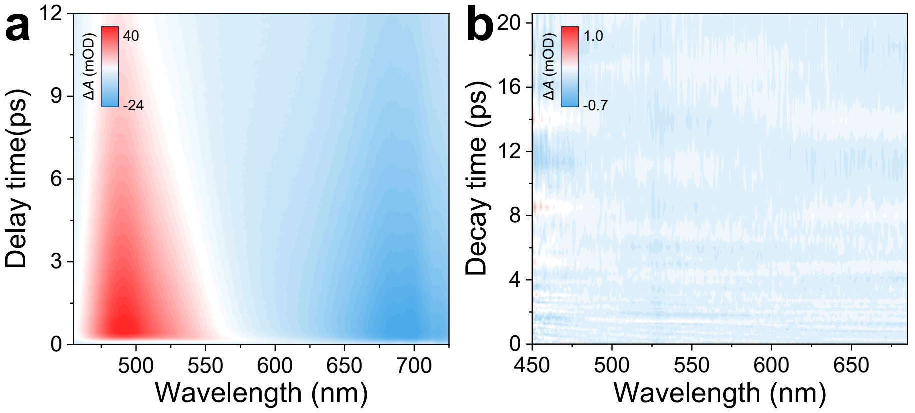

Supplementary Figure 22. TAS data after excitation at 785 nm. TAS map for **a** Au NPs and **b** TiO_2 NAs. Source data are provided as a Source Data file.

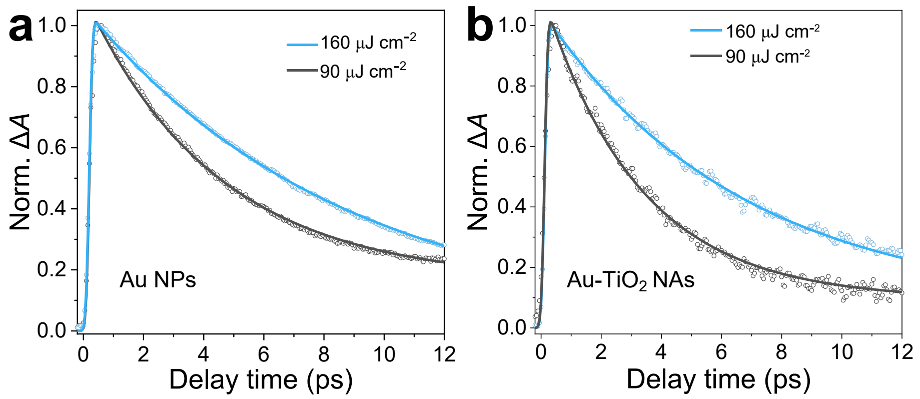

Supplementary Figure 23. The dynamics of laser heating. Dynamics of **a** Au NPs and **b** Au-TiO_2 NAs with laser energy densities of 160 μJ cm^{-2} and 90 μJ cm^{-2}, extracted at the maximum winglet peak and pumped at 785 nm. The relaxation dynamics slow down with increasing laser energy densities, consistent with previous standard "solid-state" integral model and experiments[14-16]. Source data are provided as a Source Data file.

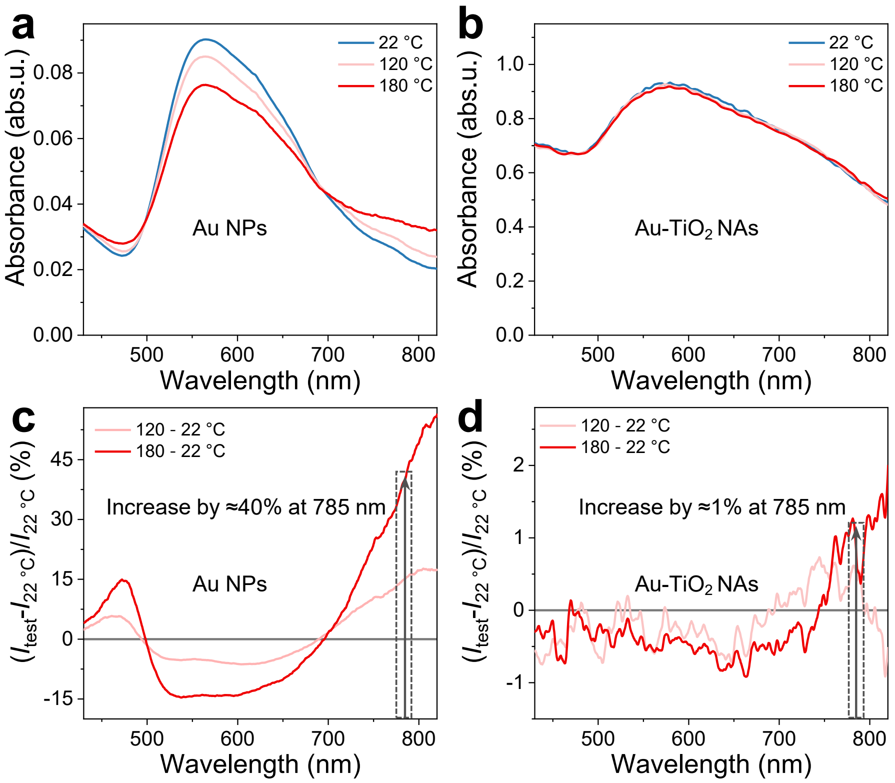

Supplementary Figure 24. The in situ steady-state absorption spectra of **a** Au NPs and **b** Au-TiO_2 NAs, tested at room temperature, 120 °C and 180 °C; Differential-absorption spectra of **c** Au NPs and **d** Au-TiO_2 NAs relative to room-temperature absorptance, calculated from the date of **a** and **b**. The absorption of Au nanoparticles at 785 nm increases by ≈40% at 180 °C compared to 22 °C, consistent with previous temperature-dependent permittivity models[17-20]. Source data are provided as a Source Data file.

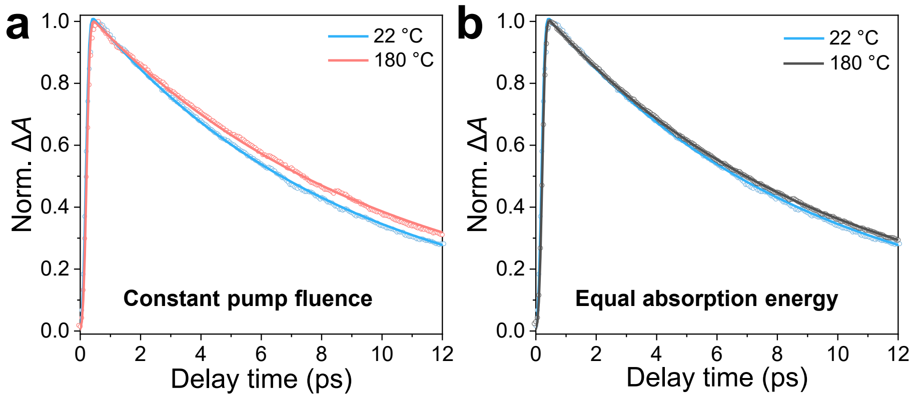

Supplementary Figure 25. a Dynamics of Au NPs at 22 ℃ and 180 ℃ with constant pump fluence, and Au NPs at 180 ℃ exhibited obviously slower $\tau_{e\text{–}ph}$ (9.7 ± 0.6 ps) than Au NPs at 22 ℃ (8.6 ± 0.5 ps); **b** Dynamics of Au NPs at 22 ℃ and 180 ℃ with equal absorption energy, and Au NPs at 180 ℃ exhibited slightly slower $\tau_{e\text{–}ph}$ (9.1 ± 0.5 ps) than Au NPs at 22 ℃ (8.6 ± 0.5 ps); All kinetics were extracted at the maximum winglet peak of Au, pumped at 785 nm. Source data are provided as a Source Data file.

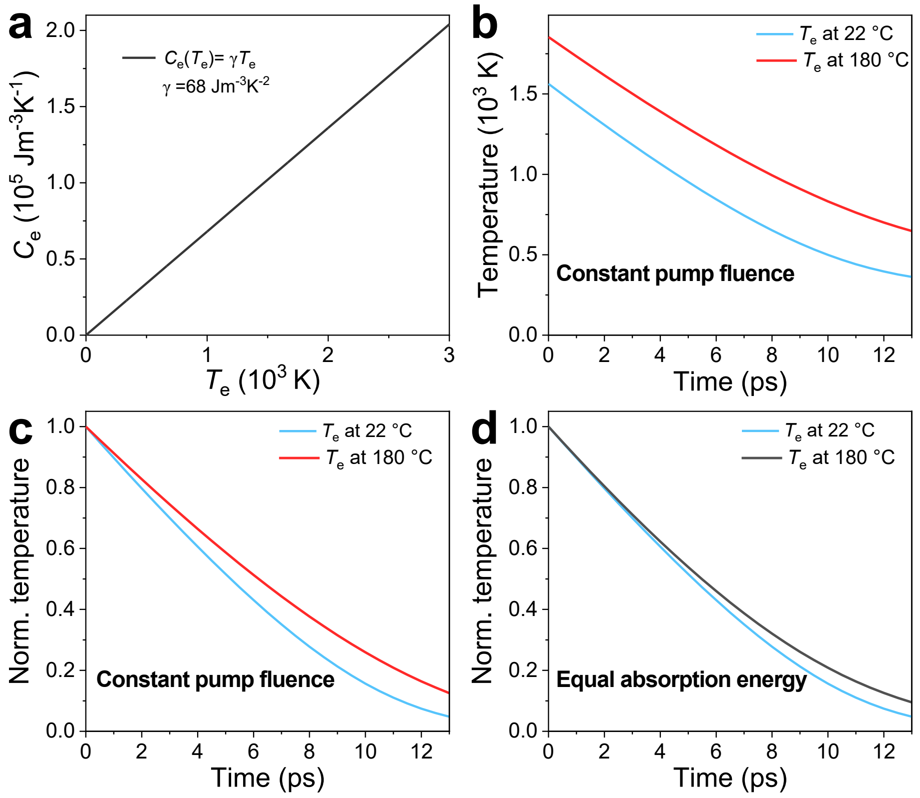

Supplementary Figure 26. a The heat capacity of the electrons as a function of electron temperature; **b** With constant pump fluence, two-temperature model simulations showing the temporal evolution of electron temperatures in Au NPs at 22 °C and 180 °C; and **c** the corresponding normalized curves of the data in **b**; **d** With equal absorption energy, two-temperature model simulations showing the normalized temporal evolution of electron temperatures in Au NPs at 22 °C and 180 °C. Norm. temperature represents the normalized electron temperature of the two-temperature model.

Supplementary Note 5. Analysis of the lengthened $\tau_{e\text{–}ph}$ in Au NPs at higher temperatures.

The well-established two-temperature model[21-24] was used to rationalize the slow down relaxation of Au NPs at 180 °C (Supplementary Fig. 25a). We simulated the C_e–T_e relationship, as shown in Supplementary Fig. 26a. With constant pump fluence, the simulated $\tau_{e\text{–}ph}$ of Au NPs was 9.8 ps at 180 °C and 8.3 ps at 22 °C (Supplementary Fig. 26b, 26c), representing a 18% increase. This trend agrees with our experimental observations (Supplementary Fig. 25a).

To isolate the influence of lattice temperature on the $\tau_{e\text{–}ph}$ in Au NPs, we performed supplementary dynamics experiments with equal absorption energy (Supplementary Fig. 25b). By adjusting the pump intensity, we obtained an equal absorption energy density of of 160 μJ cm^{-2} across temperatures. The $\tau_{e\text{–}ph}$ for Au NPs exhibits a slight increase at 180 °C (9.1 ± 0.5 ps) relative to 22 °C (8.6 ± 0.5 ps), which aligns with established experimental observations[21]. With equal absorption energy, the two-temperature model yields $\tau_{e\text{–}ph}$ values of 8.3 ps at 22 °C and 9.0 ps at 180 °C (Supplementary Fig. 26d), a trend that agrees with our experimental observations.

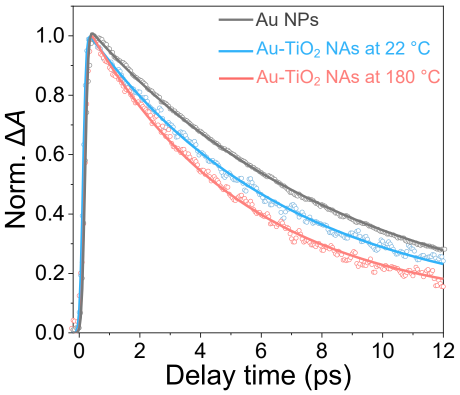

Supplementary Figure 27. Dynamics of Au NPs at 22 ℃, Au-TiO_2 NAs at 22 ℃ and 180 ℃, extracted at the maximum winglet peak of Au, pumped at 785 nm. Au NPs exhibited $\tau_{e\text{–}ph}$ of 8.6 ± 0.5 ps. Au-TiO_2 NAs at 180 ℃ exhibited faster $\tau_{e\text{–}ph}$ (5.2 ± 0.3 ps) than that at room temperature (6.6 ± 0.4 ps). Source data are provided as a Source Data file.

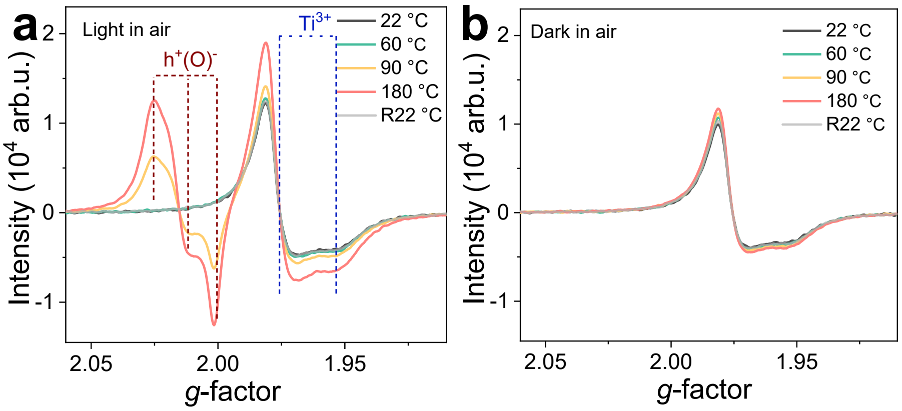

Supplementary Figure 28. The in situ EPR spectra of Au-TiO_2 NAs at different temperatures under **a** 785 nm laser irradiation and **b** dark conditions in the air atmospheres, and R22 °C represents the in-situ cooling to 22 °C. Source data are provided as a Source Data file.

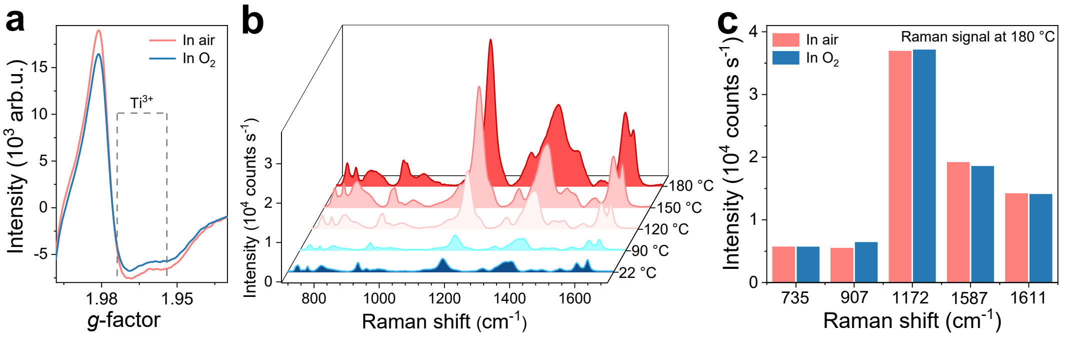

Supplementary Figure 29. a The decrease of Ti^{3+} on Au-TiO_2 NAs under 785 nm laser irradiation after adding oxygen at 180 °C; **b** The TI-SERS spectra recorded from CV (10^{-7} M) on Au-TiO_2 NAs with temperature increased from 22 to 180 °C in the O_2 atmosphere. An external mechanical pump and mass flow controllers were used to regulate the flow rates of O_2 at 40 ml/min to ensure the oxygen atmosphere; **c** The intensity comparison of the corresponding different Raman signal peaks at 180 °C in the air and oxygen atmosphere. Source data are provided as a Source Data file.

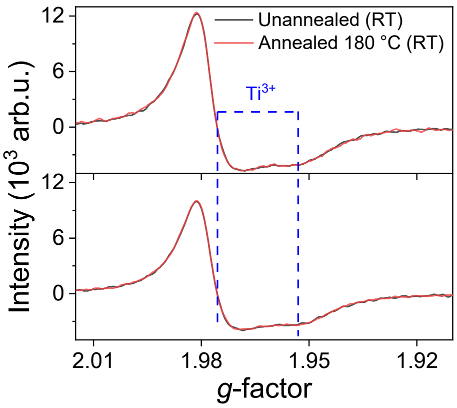

Supplementary Figure 30. The comparison of EPR spectra of 180 °C annealed (red) and unannealed (black) Au-TiO_2 NAs under 785 nm laser irradiation and dark (The spectra were obtained in an air atmosphere). The EPR spectra show the peak positions and signal amplitude of the Au-TiO_2 nanoarrays after heat treatment at 180 °C exhibit no significant changes compared with the untreated sample. Source data are provided as a Source Data file.

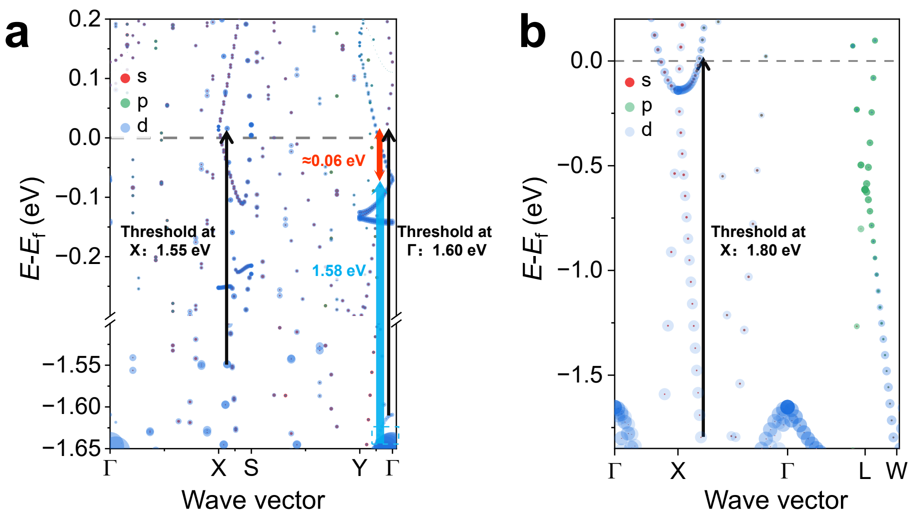

Supplementary Figure 31. The calculated fatband structures for Au component in the Au-TiO_2 NAs, and the blue dashed box represents the initial state range where transitions can occur at high temperatures; **b** The calculated fatband structures for Au bulk; The diameters of the circles represent the magnitude of density of states (DOS).

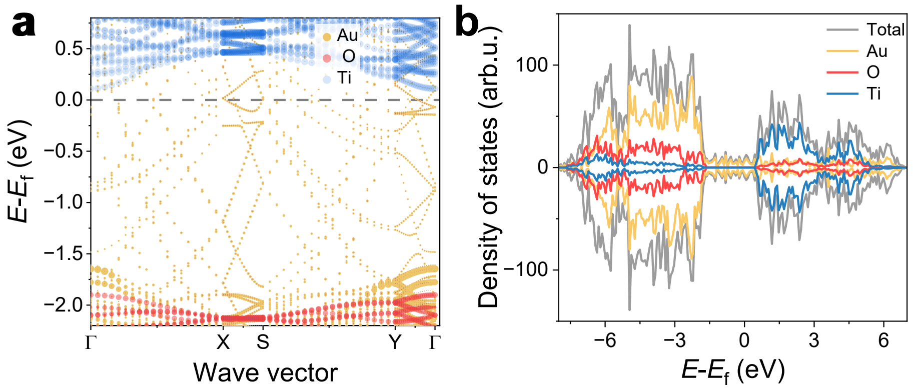

Supplementary Figure 32. The calculated **a** band structures and **b** density of states for Au-TiO_2 NAs.

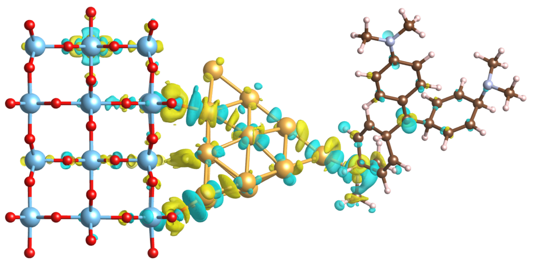

Supplementary Figure 33. Charge-density difference distribution of Au-TiO_2 with CV molecules with Au cluster without additional electrons (neutral chemisorption).

Supplementary Note 6. First-principles analysis.

We obtained the fatband structures and density of states through the first-principles calculation, as shown in Supplementary Figures 31–32. As proposed by Rosei et al.[25], on increasing the temperature, the region between the Fermi surface and the constant energy surface E = $-4K_BT$ experiences a decrease of electron population, thereby providing unoccupied states for the transition of low-energy d-band electrons. An increase in temperature from 22 °C to 180 °C (ΔT = 158 °C)

leads to an energy shift of ΔE = 0.06 eV. Subsequently, to elucidate the mechanism of high-temperature-induced interfacial hot holes, we employed first-principles calculations of the Au-TiO_2 heterostructure interface and computed its fatband structures. The band structure of the Au component in the Au-TiO_2 heterostructure shows an interband transition threshold of ≈1.55 eV near the X point (Supplementary Fig. 31a). Therefore, under 785 nm (1.58 eV) laser excitation, the energy is sufficient to trigger the interband transition at the X point. However, the fatband structure exhibits a low density of states at the X point, indicating a low transition probability. In contrast, our calculation shows that, the transition near the Γ point has a threshold of 1.60 eV coupled with a high electronic density of states. Thus, with the assistance of 0.06 eV thermal energy, the 785 nm laser excitation energy of 1.58 eV is sufficient (Supplementary Fig. 31a) to open the interband transition channel at the Γ point (1.60 eV), significantly enhancing the hot hole yield. Besides, we evaluated the change in the number of high-energy hot holes in d-band based on the fatband structures. We extracted the density of states values from the calculated results for transitions at -1.55 eV near the X point and in the range of -1.60 to -1.64 eV near the Γ point. The results indicate that the generation of high-energy hot holes could be enhanced by ≈20 times at 180 °C compared to 22 °C.

We also calculated the band structure of pure Au, which revealed an interband transition threshold of ≈1.80 eV near the X point (Supplementary Fig. 31b), in agreement with established literature[26-28]. The calculated interband transition energies of Au and the Au-TiO_2 heterostructure are 1.80 and 1.55 eV, indicating a 0.25 eV reduction for Au in the heterostructure. This reduction also benefits the generation of hot holes under 785 nm laser excitation.

First-principles density functional theory calculation was employed to gain further insight into the PICT process of TI-SERS system (Fig. 3e, Supplementary Fig. 33). The adsorption energy of the stable Au-TiO_2 with CV molecules system was calculated as -1.84 eV. Au cluster was charged with additional electron to simulate the generation of plasmon-induced large hot electrons population at high temperature[29,30]. A larger amount of electronic charge (2.13 $|e^-|$) is transferred from the Au to CV molecule when Au cluster was charged with additional electron compared with the corresponding neutral chemisorption (0.47 $|e^-|$)，which showed efficient electron transfer from Au NPs to CV molecules in the TI-SERS system.

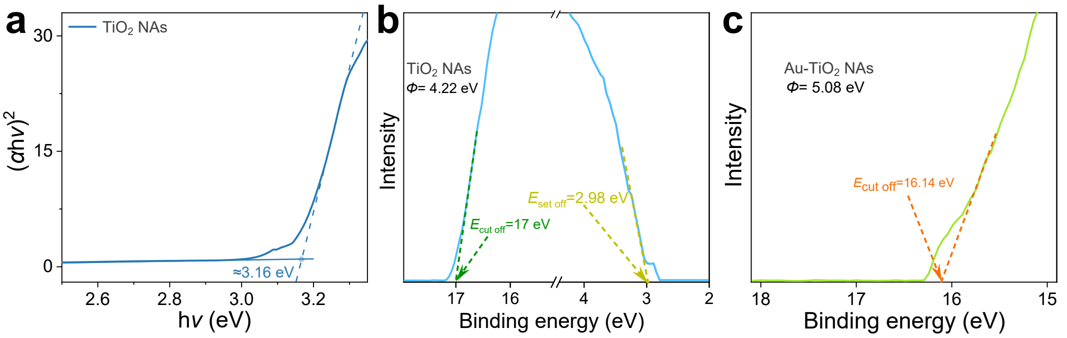

Supplementary Figure 34. a Tauc plots for the TiO_2 NAs. UPS spectra measured on **b** original TiO_2 NAs and **c** Au-TiO_2 NAs. Source data are provided as a Source Data file.

Supplementary Note 7. Band Structure Analysis.

The energy band diagram of Au-TiO_2 NAs was calculated from Tacu equation and the UPS photoemission spectra results (Supplementary Fig. 34). As shown in Supplementary Fig. 34, the typical secondary-electron cut-off energies of the TiO_2 NAs and Au-TiO_2 NAs are 17 and 16.14 eV, thereby the Fermi level (E_f) of Au-TiO_2 NAs is lower than that of TiO_2 NAs. It can be calculated that the E_f of original TiO_2 is at −4.22 eV. As displayed in Supplementary Fig. 34, the $E_{set\text{-}off}$ value of Au-TiO_2 NAs is 2.98 eV, thus the E_{VB} can be determined as -7.2 eV. Combined with their bandgap's values, the E_{CB} for TiO_2 can be determined to -4.04 eV relative to vacuum level. Similarly, the full photoemission spectrum of Au-TiO_2 NAs shows that the E_f is at −5.08 eV, which is consistent with the E_f value of Au reported previously[31,32], showing further evidence about the interface Schottky property of the Au-TiO_2 NAs[33]. Therefore, using the difference between the E_{CB} and the work function[34], we could calculate that the value of Schottky barrier height (SBH) at the interface of TiO_2 and Au was about 1.0 eV, which further demonstrated that the excitation wavelength of 785 nm (1.58 eV) is sufficient to cause a photo-induced CT contribution from Au to TiO_2.

Moreover, the highest occupied molecular orbit of CV is at -6.0 eV and the lowest unoccupied molecular orbit (LUMO) is at -4.1 eV[35]. Combining these results, we delineated the band structure of Au-TiO_2 NAs−CV molecules system. The excitation energy required for direct electron transitions of CV molecules (HUMO to LUMO) are 1.9 eV, while the excitation energy provided by the 785 nm laser is only 1.58 eV, which means that the possible directly CT process in CV is excluded. However, the laser energy is sufficient to excite direct electronic transitions from the fermi level of Au to the LUMO of CV (μ_{PICT1} 0.98 eV).

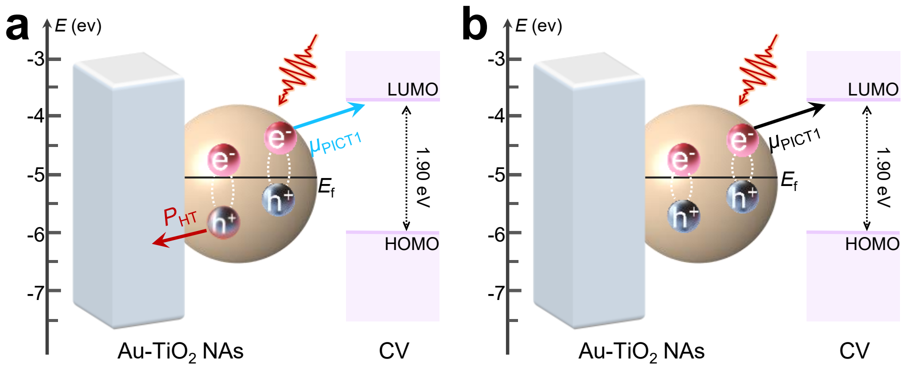

Supplementary Figure 35. Schematic diagram illustrating the CT between the Au-TiO_2 NAs substrate and CV molecule at **a** high temperature and **b** room temperature, with the excitation of 785 nm laser.

Supplementary Note 8. Photoinduced charge transfer between Au-TiO_2 NAs and CV at high and room temperatures.

As showed in Supplementary Fig. 35a, under the combined action of high temperature and light, the switch of hot electron-hole pairs from deep below the Au Fermi level excitation can be turned on. Although these hot electrons from deep below the Au Fermi level cannot be directly transferred to CV molecules, these hot holes can be transferred to TiO_2 (P_{HT}). The P_{HT} process can inhibit the recombination of hot electron-hole pairs and promote PICT. However, at room temperature, 785 nm light cannot excite the deep level electron-hole pairs (Supplementary Fig. 35b), the hot electrons on the Au particles only come from the shallow level electron-hole pairs excited by light. While the energy of these hot holes is not enough to transfer, resulting in increased electron-hole recombination and a smaller number of hot electrons, which is not conducive to SERS activity.

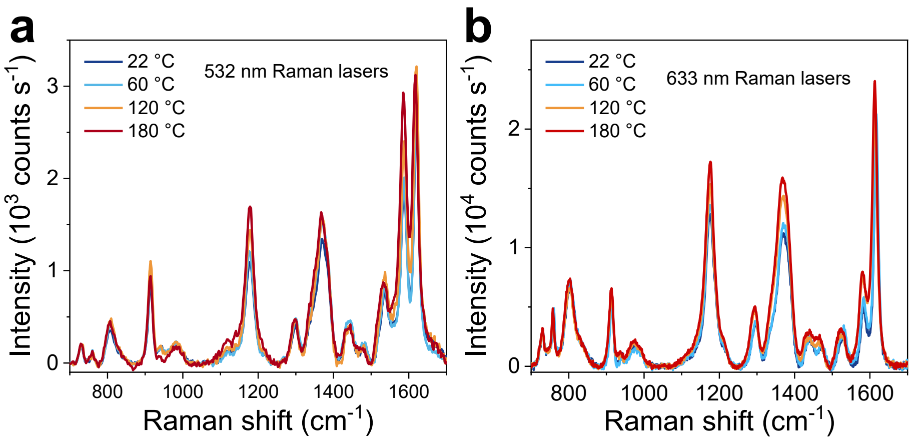

Supplementary Figure 36. The TI-SERS spectra recorded from CV (10^{-7} M) on Au-TiO_2 NAs with temperature increased from 22 °C to 180 °C using high-energy **a** 532 nm and **b** 633 nm Raman lasers, which can excite hot hole injection at Au NPs with their own energy. No significant TI-SERS enhancement was observed at high temperature for both 532 nm and 633 nm Raman lasers. It indicates that the high temperatures are not sufficient to affect the charge transfer path of the system when the energy of the photons is enough to open the hot holes transfer channel. Source data are provided as a Source Data file.

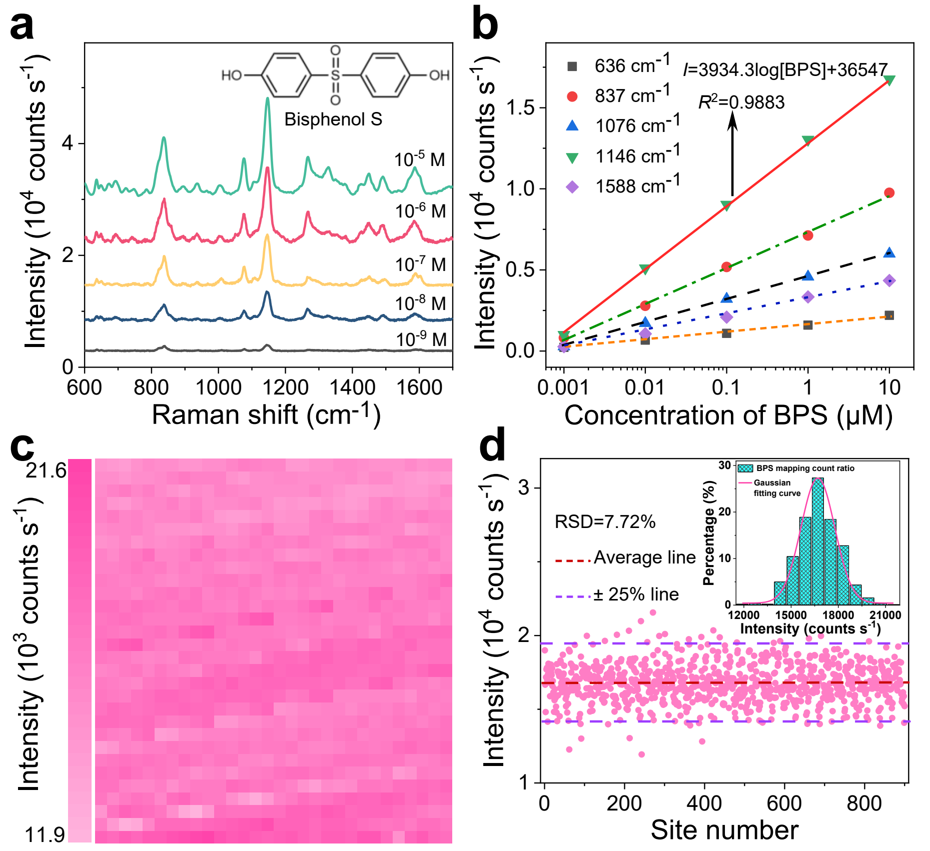

Supplementary Figure 37. Detection of bisphenol S (BPS). **a** The SERS spectra of BPS molecules with concentrations of 10^{-5}– 10^{-9} M, and the inset shows the structures of the BPS molecules; **b** The variations of Raman peak intensities at 636, 837, 1076, 1146, and 1588 cm^{-1} versus different BPS molecular concentrations. It shows a reasonable linear relationship in the logarithmic scale: I=3934.3log[BPS]+36547 (units for both constants are counts s^{-1}); **c** The spatial mapping of Raman peak intensity of BPS molecules (10^{-5} M) at 1146 cm^{-1} was performed at 900 random spots on Au-TiO_2 NAs-based substrates; **d** The scatter chart of the measured data points and the inset shows the Gaussian distribution of the measured data points. Source data are provided as a Source Data file.

Supplementary Table 2. The Comparison of analytical performance of SERS substrate with other sensors for detecting BPS reported in literature.

Analytical method	Substrate	Molecule	Detection limit	Ref.
SERS	Au@CTSA	BPS	1×10^{-6} M	Li et al.,[36]
SERS	Ag NPs-CD-SH	BPS	1×10^{-6} M	Roschi et al.,[37]
Electrochemical	AuAgPt-PCD-GO	BPS	8×10^{-9} M	Ye et al.,[38]
Electrochemical	1T&2H-MoS_2/CNTs-NH_2	BPS	2×10^{-8} M	Zhang et al.,[39]
SERS	Au-TiO_2 NAs	BPS	1×10^{-9} M	This work

Supplementary Note 9. Detecting BPS at 180 °C.

As a raw material for the production of plastics, BPS is widely found in kinds of plastic products, such as food containers, water pipes, compact discs, eyeglasses, protective equipment for sports, and various daily life products[36-42]. Tremendous damages to human endocrine, reproductive, digestive, respiratory, and central nervous systems will be caused when long-term exposure to

BPS[36-42]. What's more, numerous works have shown that plastics can decompose and release a large amount of BPS at high temperatures, which can be migrated from packaging to food and pose a huge threat to human safety[40,41]. Therefore, it is necessary to develop a SERS sensor to ultra-sensitively monitor the content of BPS at high temperatures, which could help to evaluate the environmental and human health impacts of the ubiquitous BPS and facilitate the rational design and manufacturing of safe plastics. Here, the corresponding TI-SERS analyses of BPS molecules with different concentrations (10^{-5}–10^{-9} M) were performed at 180 °C based on prepared Au-TiO_2 NAs. As shown in Supplementary Fig. 37a, the dominating characteristic bands of BPS molecules are all clearly detected in Raman spectra, which are consistent with previous works[36,37,42]. The detection limit of BPS molecules can be achieved at a low concentration of 10^{-9} M (nanomole, nM) level, which is superior to many previous studies as illustrated in Supplementary Table 2 and also significantly exceeds the maximum of residue limit (≈2×10^{-7} M) stipulated by the European Commission Regulation[37], indicating the excellent performance of the established TI-SERS system. Subsequently, to investigate the ability of quantitative detection, the variations of several Raman peak intensities of BPS molecules versus the corresponding concentrations in logarithmical scale are separately illustrated in Supplementary Fig. 37b. It can be seen that all of these plots exhibit well-defined linear relationships, which can sensitively respond to slight variations in concentration. Additionally, the uniformity of Au-TiO_2 NAs substrate is also investigated using the spatial mappings (300×300 μm) of the Raman peak intensities at 1146 cm^{-1}. As clearly depicted in Supplementary Fig. 37c–d, the Raman signals of the BPS molecules are uniform and repeatable over a large area. And the corresponding RSD values at 1146 cm^{-1} is calculated at only about 7.72% (average intensity of ≈16753 a.u), which further demonstrates the excellent uniformity of the Au-TiO_2 NAs substrate. Moreover, the Gaussian distribution of the data points further confirms the validity of the experimental data.

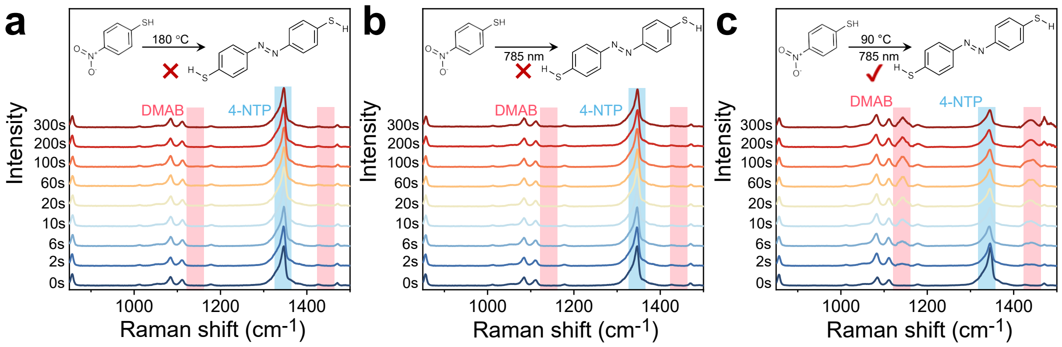

Supplementary Figure 38. The normalized time-dependent SERS spectra of 4-NTP dimerization into DMAB based on Au-TiO_2 NAs with **a** Thermally driven (temperature of 180 °C), **b** Plasmon-driven (785 nm laser powers of 0.05 mW cm^{-2}), and **c** Thermally assisted plasmons driven (785 nm laser powers of 0.05 mW cm^{-2} and temperature of 90 °C) at different time intervals were recorded. The relative intensities of the spectral lines in **c** are normalized with the peak at 1076 cm^{-1} for comparison[43]. Source data are provided as a Source Data file.

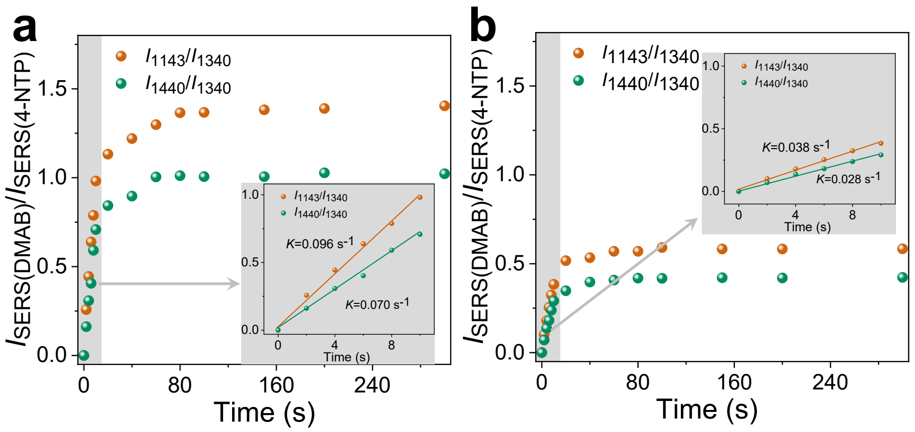

Supplementary Figure 39. The rate constants of two different bands of Au-TiO_2 NAs catalyzed thermally assisted plasmon driven catalytic reaction of 4-NTP dimerizing into DMAB.The laser powers of catalysis are contained at 0.05 mW cm^{-2} and temperature at **a** 180 °C, **b** 90 °C. The reaction rate K for the conversion of 4-NTP to DMAB can be characterized by the slope of $I_{SERS(DMAB)}/I_{SERS(4\text{-}NTP)}$, according to previous work[44]. Source data are provided as a Source Data file.

Supplementary references

1 Wang, Y. *et al.* Facile fabrication of Ag/graphene oxide/TiO_2 nanorod array as a powerful substrate for photocatalytic degradation and surface-enhanced Raman scattering detection. *Appl. Catal. B*, **252,** 174-186 (2019).

2 Verbruggen, S. W. *et al.* Plasmonic gold–silver alloy on TiO_2 photocatalysts with tunable visible light activity. *Appl. Catal. B*, **156,** 116-121 (2014).

3 Halas, N. J., Lal, S., Chang, W. S., Link, S. & Nordlander, P. Plasmons in strongly coupled metallic nanostructures. *Chem. Rev.*, **111,** 3913-3961 (2011).

4 Liu, Z. *et al.* Piezoelectric - effect - enhanced full - spectrum photoelectrocatalysis in p – n heterojunction. *Adv. Funct. Mater.*, **29,** 1807279 (2019).

5 Cho, I. S. *et al.* Branched TiO_2 nanorods for photoelectrochemical hydrogen production. *Nano Lett.*, **11,** 4978-4984 (2011).

6 Liu, G. *et al.* Boosting photocatalytic nitrogen reduction to ammonia by dual defective -CN and K-doping sites on graphitic carbon nitride nanorod arrays. *Appl. Catal. B*, **317,** 121752 (2022).

7 Ma, H. *et al.* Design of a thermally stable and highly active SERS optical sensor for the ultrasensitive detection of dye molecules at high-temperature. *Opt. Mater. Express*, **11,** 2001-2015 (2021).

8 Fularz, A., Almohammed, S. & Rice, J. H. Oxygen incorporation-induced SERS enhancement in silver nanoparticle-decorated ZnO nanowires. *ACS Appl. Nano Mater.*, **3,** 1666-1673 (2020).

9 Ben-Jaber, S. *et al.* Photo-induced enhanced Raman spectroscopy for universal ultra-trace detection of explosives, pollutants and biomolecules. *Nat. Commun.*, **7,** 12189 (2016).

10 Fularz, A., Almohammed, S. & Rice, J. H. Controlling plasmon-induced photocatalytic redox reactions on WO_3 nanowire/AgNPs substrates via defect engineering. *J. Phys. Chem. C*, **124,** 25351-25360 (2020).

11 Wang, L. *et al.* Enhancement of pyridine derivatives containing symmetrical substituents on the photocatalytic degradation of phenol and antibiotics by Er-Fe-TiO_2. *Chem. Eng. J.*, **410,** 128319 (2021).

12 Makuła, P., Pacia, M. & Macyk, W. How to correctly determine the band gap energy of modified semiconductor photocatalysts based on UV–Vis spectra. *J. Phys. Chem. Lett.*, **9,** 6814-6817 (2018).

13 Ji, R. *et al.* Perovskite phase heterojunction solar cells. *Nat. Energy*, **7,** 1170-1179 (2022).

14 Del Fatti, N. *et al.* Nonequilibrium electron dynamics in noble metals. *Phys. Rev. B*, **61,** 16956-16966 (2000).

15 Saavedra, J. R. M., Asenjo-Garcia, A. & García de Abajo, F. J. Hot-electron dynamics and thermalization in small metallic nanoparticles. *ACS Photonics*, **3,** 1637-1646 (2016).

16 Tagliabue, G. *et al.* Ultrafast hot-hole injection modifies hot-electron dynamics in Au/p-GaN heterostructures. *Nat. Mater.*, **19,** 1312-1318 (2020).

17 Reddy, H. *et al.* Temperature-dependent optical properties of single crystalline and polycrystalline silver thin films. *ACS Photonics*, **4,** 1083-1091 (2017).

18 Shen, P. *et al.* Temperature- and roughness-dependent permittivity of annealed/unannealed gold films. *Opt. Express*, **24,** 19254-19263 (2016).

19 Gurwich, I. & Sivan, Y. Metal nanospheres under intense continuous-wave illumination: A unique case of nonperturbative nonlinear nanophotonics. *Phys. Rev. E*, **96,** 012212 (2017).

20 Ferrera, M., Magnozzi, M., Bisio, F. & Canepa, M. Temperature-dependent permittivity of silver

and implications for thermoplasmonics. *Phys. Rev. Mater.*, **3,** 105201 (2019).

21 Groeneveld, R. H. M., Sprik, R. & Lagendijk, A. Femtosecond spectroscopy of electron-electron and electron-phonon energy relaxation in Ag and Au. *Phys. Rev. B*, **51,** 11433-11445 (1995).

22 Zhou, M. *et al.* Evolution from the plasmon to exciton state in ligand-protected atomically precise gold nanoparticles. *Nat. Commun.*, **7,** 13240 (2016).

23 D, A. C. & Ray, A. Two-temperature model for ultrafast melting of Au-based bimetallic films interacting with single-pulse femtosecond laser: Theoretical study of damage threshold. *Phys. Rev. B*, **107,** 195402 (2023).

24 Hu, M. & Hartland, G. V. Heat dissipation for Au particles in aqueous solution: relaxation time versus size. *J. Phys. Chem. B*, **106,** 7029-7033 (2002).

25 Rosei, R. Temperature modulation of the optical transitions involving the fermi surface in Ag: Theory. *Phys. Rev. B*, **10,** 474-483 (1974).

26 Beversluis, M. R., Bouhelier, A. & Novotny, L. Continuum generation from single gold nanostructures through near-field mediated intraband transitions. *Phys. Rev. B*, **68,** 115433 (2003).

27 Brown, A. M., Sundararaman, R., Narang, P., Goddard, W. A., III & Atwater, H. A. Nonradiative pasmon decay and hot carrier dynamics: effects of phonons, surfaces, and geometry. *ACS Nano*, **10,** 957-966 (2016).

28 Karaman, C. O., Bykov, A. Y., Kiani, F., Tagliabue, G. & Zayats, A. V. Ultrafast hot-carrier dynamics in ultrathin monocrystalline gold. *Nat. Commun.*, **15,** 703 (2024).

29 Hu, C. *et al.* Surface plasmon enabling nitrogen fixation in pure water through a dissociative mechanism under mild conditions. *J. Am. Chem. Soc.*, **141,** 7807-7814 (2019).

30 Yao, K. *et al.* Plasmon-induced trap filling at grain boundaries in perovskite solar cells. *Light:Sci. Appl.*, **10,** 219 (2021).

31 Pham, T., Li, G., Bekyarova, E., Itkis, M. E. & Mulchandani, A. MoS_2-based optoelectronic gas sensor with sub-parts-per-billion limit of NO_2 gas detection. *ACS Nano*, **13,** 3196-3205 (2019).

32 Xue, Z. *et al.* Electrochemical reduction of N_2 into NH_3 by donor–acceptor couples of Ni and Au nanoparticles with a 67.8% faradaic efficiency. *J. Am. Chem. Soc.*, **141,** 14976-14980 (2019).

33 Park, J. Y., Lee, H., Renzas, J. R., Zhang, Y. & Somorjai, G. A. Probing hot electron flow generated on Pt nanoparticles with Au/TiO_2 schottky diodes during catalytic CO oxidation. *Nano Lett.*, **8,** 2388-2392 (2008).

34 Zhang, Z. & Yates, J. T. Band bending in semiconductors: chemical and physical consequences at surfaces and interfaces. *Chem. Rev.*, **112,** 5520-5551 (2012).

35 Dharmalingam, P., Venkatakrishnan, K. & Tan, B. An atomic-defect enhanced Raman scattering (DERS) quantum probe for molecular level detection–breaking the SERS barrier. *Appl. Mater. Today*, **16,** 28-41 (2019).

36 Li, R. *et al.* Machine learning-driven 3D plasmonic cellulose sensor for in situ rapid SERS detection of bisphenol compounds in water sample. *Talanta*, **265,** 124917 (2023).

37 Roschi, E. *et al.* Surface-enhanced Raman spectroscopy for bisphenols detection: toward a better understanding of the analyte–nanosystem interactions. *Nanomaterials*, **11,** 881 (2021).

38 Ye, Z., Wang, Q., Qiao, J., Ye, B. & Li, G. Simultaneous detection of bisphenol A and bisphenol S with high sensitivity based on a new electrochemical sensor. *J. Electroanal. Chem.*, **854,** 113541 (2019).

39 Zhang, L. *et al.* Sensitive and direct electrochemical detection of bisphenol S based on 1T&2H-MoS_2/CNTs-NH_2 nanocomposites. *New J. Chem.*, **46,** 8203-8214 (2022).

40 Li, Y. *et al.* Photoaging of baby bottle-derived polyethersulfone and polyphenylsulfone microplastics and the resulting bisphenol S release. *Environ. Sci. Technol.*, **56,** 3033-3044 (2022).

41 García-Córcoles, M. T. *et al.* Determination of bisphenols with estrogenic activity in plastic packaged baby food samples using solid-liquid extraction and clean-up with dispersive sorbents followed by gas chromatography tandem mass spectrometry analysis. *Talanta*, **178,** 441-448 (2018).

42 Ullah, R. & Wang, X. Raman spectroscopy of bisphenol 'S' and its analogy with bisphenol 'A' uncovered with a dimensionality reduction technique. *J. Mol. Struct.*, **1175,** 927-934 (2019).

43 Zhu, Y., Tang, H., Wang, H. & Li, Y. In Situ SERS monitoring of the plasmon-driven catalytic reaction by using single Ag@Au nanowires as substrates. *Anal. Chem.*, **93,** 11736-11744 (2021).

44 Tian, Y. *et al.* Extra electric field-enhanced lightning rod effect in pine needle-like Au microarrays for boosting direct plasmon-driven photoelectrochemical hydrogenation reactions via in-situ SERS monitoring. *Appl. Surf. Sci.*, **578,** 152100 (2022).
