# Supplementary material for: Heat-assisted hot-hole transfer increases the surface-enhanced Raman activity of Au-TiO2 nanoarrays
Source: Nat Commun. 2026 Mar 17;17:4047. doi: 10.1038/s41467-026-70822-4 (PMC13139474; doi:10.1038/s41467-026-70822-4)
Supplement: Supplementary file 2 — Description of Additional Supplementary Files [file 41467_2026_70822_MOESM2_ESM.pdf]

Description of Additional Supplementary Files

File Name: Supplementary Data 1

Description: Unprocessed raw data.
